# Primary ciliated muconodular papillary tumor: A rare pulmonary disease and literature review of 65 cases

**DOI:** 10.1111/1759-7714.13963

**Published:** 2021-05-07

**Authors:** Yanye Wang, Dan Wang, Jun Wang, Shikang Zhao, Dian Ren, Gang Chen, Qiuhui Wang, Dongbo Xu, Song Xu

**Affiliations:** ^1^ Department of Lung Cancer Surgery Tianjin Medical University General Hospital Tianjin China; ^2^ Tianjin Key Laboratory of Lung Cancer Metastasis and Tumor Microenvironment Lung Cancer Institute, Tianjin Medical University General Hospital Tianjin China; ^3^ Department of Pathology Tianjin Medical University General Hospital Tianjin China; ^4^ Department of General Surgery Tianjin Teda Hospital Tianjin China; ^5^ Department of Radiology Tianjin Medical University General Hospital Tianjin China

**Keywords:** bronchiolar adenoma (BA), ciliated muconodular papillary tumor (CMPT), PD‐L1, sequencing, surgery

## Abstract

A ciliated muconodular papillary tumor (CMPT) or bronchiolar adenoma (BA) is a rather rare and unique type of lung tumor characterized by tripartite cellular components with a papillary‐predominant structure including ciliated columnar cells, mucinous cells, and basal cells. Here, we present the case of a 64‐year‐old woman who was diagnosed with CMPT in our center. In addition to reporting the clinicopathological characteristics of this case, we also conducted whole exome sequencing (WES) to explore the underlying mechanism. According to current evidence, CMPTs tends to be benign or of low grade malignancy. However, this requires further validation.

## Graphical abstract

A ciliated muconodular papillary tumor (CMPT)/bronchiolar adenoma (BA) is a rare and rather unique type of lung tumor characterized by tripartite cellular components with a papillary‐predominant structure including ciliated columnar cells, mucinous cells, and basal cells. We present the case of a 64‐year‐old woman who was diagnosed with CMPT in our center. In addition to reporting the clinicopathological characteristics of this case, we also conducted whole exome sequencing (WES) to explore the underlying mechanism. According to the current evidence, CMPTs tends to be benign or of low grade malignancy. However, this requires further validation.

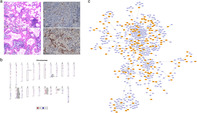

## INTRODUCTION

Ciliated muconodular papillary tumor (CMPT) is a rare pulmonary neoplasm which is considered benign or of low‐grade malignancy. However, treatment and pathogenesis are still unclear for this rare lung tumor. Here, we present a case of CMPT in a 64‐year‐old woman in whom we whole exome sequencing (WES) was conducted to explore the underlying mechanism. In addition, we discuss the clinicopathological and genetic characteristics of this rare pulmonary tumor according to the published literature.

## CASE REPORT

**FIGURE 1:**
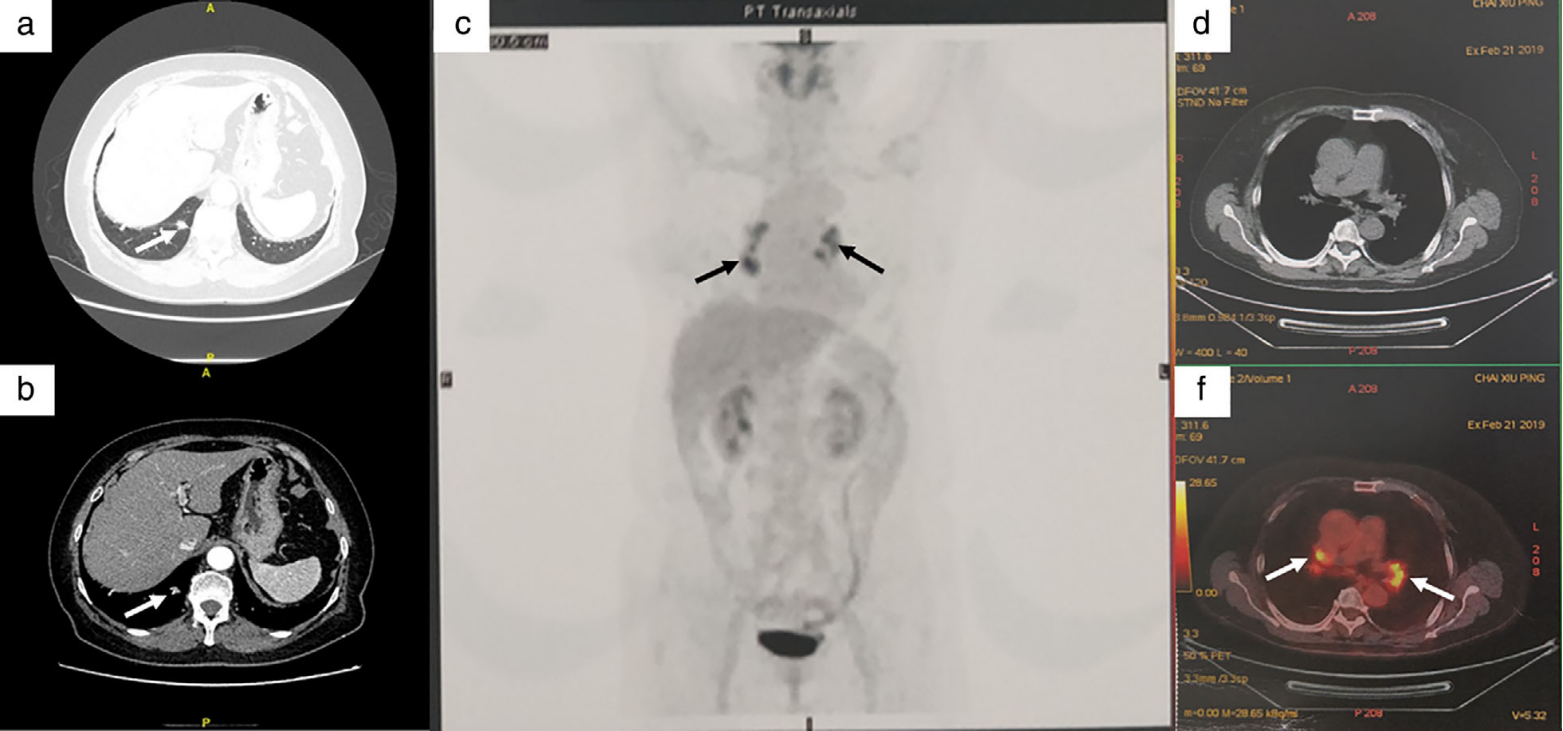
(a, b) Contrast‐enhanced computed tomography (CT) indicated an irregular solid nodule with a maximum diameter of 1.2 cm in the dorsal segment of the right lower pulmonary lobe. (c, d, e) 18F‐FDG CT‐PET showed focal hyperaccumulation within the pulmonary artery and hilum and mediastinal lymph nodes (LNs)

A nodule was detected in the right lower lobe of the lung of a 64‐year‐old woman without any symptoms during a physical examination. There was no exposure to tuberculosis. The patient's vital signs and routine blood tests did not show any particular abnormalities. CT (computed tomography) scan indicated a solid nodule 1.2 cm in diameter in the right lower lobe of the lung that invaded the pleura and had an ill‐defined margin (Figure 1(a) and (b)). Multiple enlarged lymph nodes (LNs) were observed in the hilum and mediastinum (subcarinal space, right‐sided paratracheal space and aortopulmonary window). Positron emission tomography‐CT (PET‐CT) demonstrated increased 18F‐FDG uptake in the nodule of the right lower lobe (maximum standardized uptake value [SUVmax] = 4.36), as well as in the hilar and mediastinal LNs (SUVmax = 11.4) (Figure 1(c)–(e)). No abnormalities were found in the other organs on PET‐CT. The levels of tumor markers, including squamous cell carcinoma antigen, carcinoembryonic antigen (CEA), cytokeratin‐19 (CK19) fragment, neuron‐specific enolase, and progastrin releasing peptide, were all normal.

**FIGURE 2:**
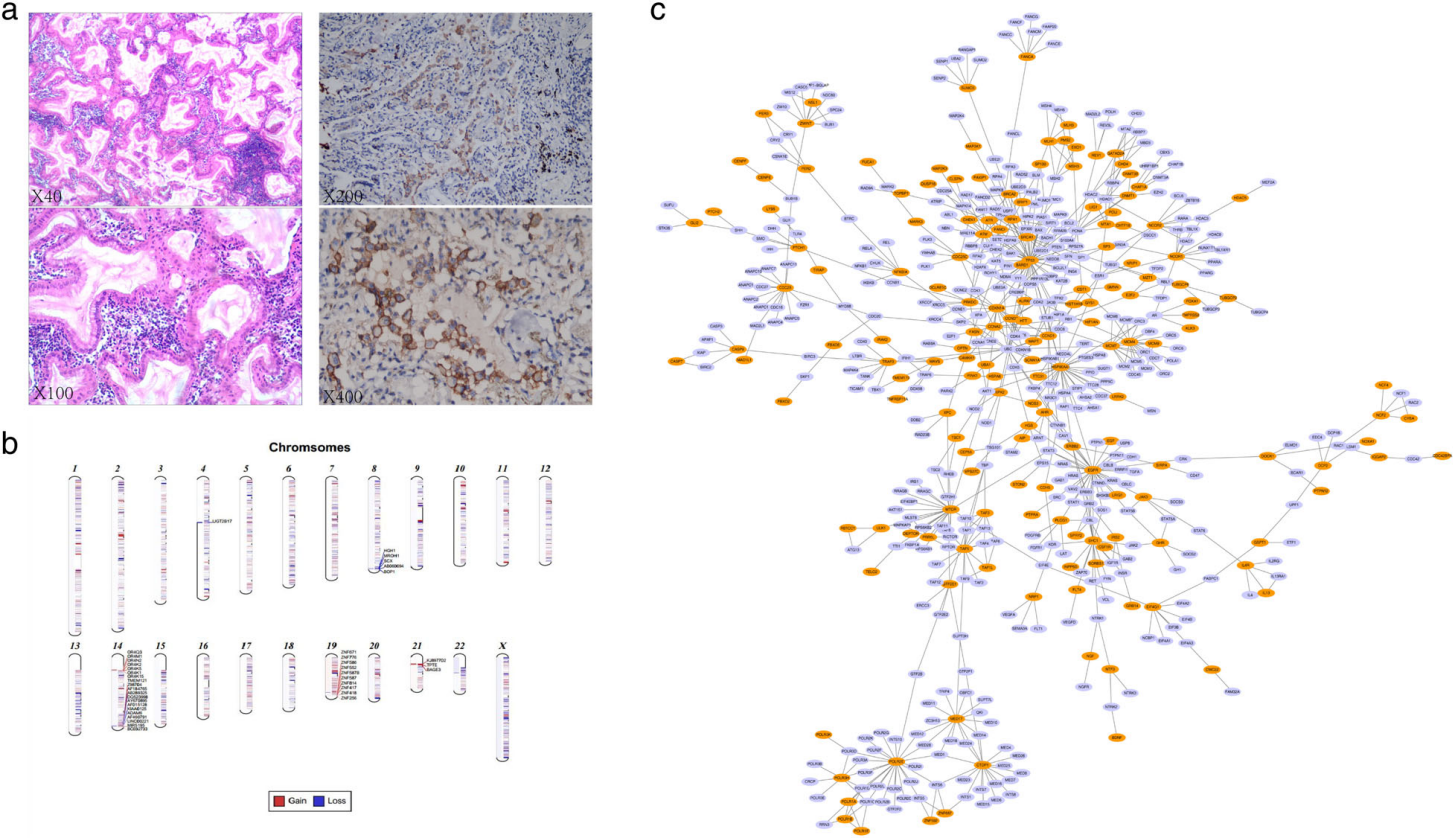
(a) Histopathological examination with hematoxylin and eosin (H&E) staining confirmed the lesions were surrounded by copious amounts of mucus and consist of ciliated columnar cells, mucous cells, and basal cells (×40, ×100). PD‐L1 expression (22C3 Darko antibody) in the solid nodule tissue (×200, ×400). (b) The patient's whole genome CNV model ideograph (red = gain, blue = loss). (c) Patient's protein–protein interaction (PPI) network complex analysis (154 upregulated in orange [upregulation] and 421 downregulated genes in blue [downregulation])

We considered that the patient may have multiple pulmonary metastases and therefore a surgical biopsy was performed through a thoracoscopic wedge resection of the right lower lobe and mediastinal LN sampling. Pathological examination showed that ciliated columnar cells were coated on the surface of the adenoid or papillary structure. The tumor cells showed no necrosis, atypia or mitosis (Figure 2(a)), and presented as a mixture of bland ciliated columnar cells, a basal cell layer and mucinous cells. The immunohistochemical profile of the ciliated columnar cells and mucous cells showed positive CK7, EMA and CEA expression, weakly positive TTF‐1 expression, and negative CDX2, villin, and vimentin expression, as well as positive CD34 and D2‐40 vascular endothelial cells. The tumor showed a low Ki‐67 proliferating index (approximately 5%). The lymph nodes from the subcarinal and right sided paratracheal space exhibited reactive hyperplasia without malignancy. Therefore, a diagnosis of CMPT was considered.

The patient was also tested using whole‐exome sequencing (WES), and a copy number variation (CNV) image was built to express the significant mutations (Figure 2(b)) and a bioinformatic analysis was performed according to the WES data (Figure 2(c)). A total of 15 somatic gene mutations were found in the analysis, but none of the known driver genes in lung cancer, such as *EGFR* and *ALK*, were identified (Table 1). We also tested for PD‐L1 expression (22C3 Darko antibody) and found a low expression (5% TPS) in the tumor tissue (Figure 2(a)). These had not metastasized to the local lymph nodes or distant sites.

**TABLE 1:** Somatic gene mutations by whole exome sequencing (WES)

Gene	Mutation_type	Amino acid and base change	Frequency (%)	Exon_rank	CHROM
TPTE2	Missense_variant	p.Tyr449Phe c.1346_1347delinsTC	9.20	19	13
SUSD2	Missense_variant	p.Gly70Val c.209_210delinsTC	9.60	2	22
CHIT1	Stop_gained	p.Val357_Trp358insTerGlyLeuGlyGlyAlaMetVal c.1049_1072dupAGGGACTGGGCGGGGCCATGGTCT	11.20	10	1
RPL14	Conservative_inframe_insertion	p.Ala159dup c.475_477dupGCT	5.70	6	3
TPTE2	Missense_variant	p.Asn447Asp c.1339A > G	8.70	19	13
RP1L1	Conservative_inframe_deletion	p.Glu1343del c.4027_4029del	8.20	4	8
RP1L1	Conservative_inframe_insertion	p.Glu1300_Val1301insGly c.3901_3902insGGG	5.60	4	8
HLX	Missense_variant	p.Gln134Pro c.401A > C	13.78	1	1
FAM155A	Missense_variant	p.Arg77Gln c.230G > A	7.28	1	13
NOP9	Disruptive_inframe_insertion	p.Glu168_Glu169dup c.504_509dup	17.35	2	14
ZNF839	Conservative_inframe_insertion	p.Gly19_Gly20dup c.55_60dup	12.93	1	14
SBK3	Missense_variant	p.Phe319Val c.955 T > G	6.05	4	19
TMEM247	Missense_variant	p.Arg129Gln c.386G > A	19.84	2	2
CACNA1D	Disruptive_inframe_deletion	p.Met7del c.20_22del	6.02	1	3
HAVCR1	Missense_variant	p.Met158Thr c.473 T > C	13.01	4	5

## DISCUSSION

In 2002, Ishikawa et al. first reported a case of CMPT that was characterized by tripartite cellular components with a papillary‐predominant structure including ciliated columnar cells, mucinous cells, and basal cells.^1^ Dr Tsao raised that bronchiolar adenoma/ciliated muconodular papillary tumor is a benign tumor during the 2020 World Conference on Lung Cancer in the fifth edition of the World Health Organization (WHO) Book on Classification of Thoracic Tumors. We conducted a comprehensive literature review, and 65 cases have been reported to date^2^, ^3^, ^4^, ^5^, ^6^, ^7^, ^8^, ^9^, ^10^, ^11^, ^12^, ^13^, ^14^, ^15^, ^16^, ^17^, ^18^ (Table 2). Approximately 44.6% (29/65) of the patients had a history of smoking. The most common location was the right lower lobe followed by the left lower lobe; tumors in the upper lung fields have also been reported. The CMPT lesions were located in the peripheral lung fields. More tumors have been observed in the lower than in the upper lobes. The average size of CMPTs reported in the literature is 11.8 mm (ranging from 4 mm to 45 mm). Therefore, appropriate surgical procedures and LN sampling were performed to clarify the diagnosis. Sublobar resection was the most common treatment for CMPT, which accounted for 69.2% of the patients with known surgical procedures (27/39 cases) from the data. However, because of the clinical rarity of this tumor, uniform diagnostic criteria are lacking. A radical resection was not appropriate, and therefore a wedge resection of the right lung lobe as well as LN sampling was performed. According to the literature, regardless of the type of surgical intervention (lobar or sublobar resection), no recurrences or metastases have been reported with follow‐up periods ranging up to 10 years.

**TABLE 2:** Summary of clinical data and detected gene mutations in previous reports and present cases

SOURCE	Cases	Age years/sex	Smoking history	Location	Size (mm)	Treatment	Follow‐up (months)	Gene mutation
Ishikawa et al.^1^	1	50/male	Yes	RUL	15	Lobectomy	120	Unknown
Harada et al.^2^	1	62/male	Yes	LLL	9	Partial resection	24	Unknown
Sato et al.^3^	2	Mean: 63 (range: 59–67)/M:F = 1:1	YES NO	RLL RUL	5 8	Partial resection Partial resection	Mean: 14 (range: 10–18)	Unknown
Hata et al.^4^	1	76/male	NO	LUL	7	Lobectomy	24	Unknown
Chuang et al.^5^	1	68/male	YES	RLL	12	Wedge resection	48	Unknown
Chu et al.^6^	1	56/male	unknown	LUL	8	Segmentectomy	5	Unknown
Kamata et al.^7^	10	Median: 61.5 (range: 56–78)/M:F = 7:3	YES = 5；NO = 5	RLL = 5; RUL = 1; LLL = 4	10 (range: 6–15)	Lobectomy = 1 Segmentectomy = 1; Wedge resection = 8	Mean: 43 (range: 2–88)	*BRAF* G606R: 2 *BRAF* V600E: 3 *EGFR*: 3 Unknown: 2
Kon et al.^8^	5	80/male	Unknown	LLL	7	Wedge resection	29	Unknown
Lau et al.^9^	1	19/female	No	RLL	13	Wedge resection	Unknown	Unknown
		67/male	Unknown	RLL	10	Wedge resection	25	Unknown
		66/male	Unknown	RLL	13	Lobectomy	14	Unknown
		73/female	Unknown	LUL	9	Wedge resection	5	Unknown
		70/female	Unknown	RLL	8	Wedge resection	24	Unknown
Liu et al.^10^	4	Median: 76 (range: 60–83) M:F = 1:3	Yes: 1 Unknown: 3	RLL RML LEFT LUL	MEAN: 7.5 (Range: 4–12)	Wedge resection: 3 Lobectomy:1	7;10 Unknown: 2	*BRAF* V600E: 1 ALK: 1
Ishikawa et al.^11^	5	Mean: 72.4 (range = 66–82)/M:F = 3:2	Yes = 3; No = 2	RUL:1 RLL:2 LLL:2	Mean: 21.6 (range: 5–45)	Lobectomy: 2 Partial resection: 3	41.6 (range: 19–58)	Unknown
Ji et al.^12^	1	59/female	No	RLL	8	Lobectomy	6	*ALK*
Taguchi et al.^13^	1	84/female	No	RLL	10	Partial resection	10	Unknown
Udo et al.^14^	4	Median: 67; M/F = 0:4	No	Unknown	Median: 11 (range: 8–25)	Lobectomy: 3; Segmentectomy:1	Unknown	*BRAF* V600E; *KRAS* G12D; *AKT* E17K
Cheung et al.^15^	1	61/male	Yes	RLL	8	Lobectomy	12	Unknown
Kashima et al.^16^	5	Mean = 67.2 (range: 57–73)/M:F = 2:3	unknown	RLL = 2 LLL = 2 LUL = 1	Mean = 11.2 (range: 8–18)	Unknown	Mean = 34.8 (range:6–72)	*BRAF* V600E:3
Chang et al. ^17^	21	Mean = 75.1 (range: 55–78)/M:F = 11:10	Yes = 15 No = 4 Unknown = 2	NA	Mean = 7.7 (range: 2–20)	Unknown	Unknown	*BRAF* V600E: 8 *EGFR*: 4 *KRAS*: 4 *HRAS*: 1 Unknown: 4
TOTAL	65	Mean = 70.1 (range: 19–83) :/M:F = 34:31	Yes: 29 No: 24 Unknown:1 2	RUL: 4 RML: 1 RLL: 19 LUL: 5 LLL: 10 Left: 1 Unknown: 4	Mean: 11.8 Range:4–45	Lobectomy:12 Partial resection (wedge resection and segment): 27 Unknown: 5	Mean: 26.1 months (range: 2–120)	*ALK*: 2 *AKT* E17K: 1 *BRAF* V600E: 16 *BRAF* G606R: 2 *EGFR*: 7 *HRAS*: 1 *KRAS*: 5 Unknown: 31

Abbreviations: LLL, left lower lobe; LUL, left upper lobe; RLL, right lower lobe; RML, right middle lobe; RUL, right upper lobe; Unknown, not available.

With regard to the pathological diagnosis of CMPT, the immunohistochemistry staining showed the positive CK7 and negative CK20 expression of the ciliated columnar cells and mucous cells. Some studies have previously reported focally weak TTF‐1 expression, while others have indicated that CMPTs have no TTF‐1 expression.^1^, ^3^, ^8^ In this case, the tumor cells expressed CK7 and weakly expressed TTF‐1; however, the mucous cells were negative for both markers; all cells generally had a low Ki‐67 proliferating index. In terms of gene mutation, these studies have identified several molecular alterations, and *BRAF* mutations are the most common gene. Kamata et al. reported five cases of CMPTs with mutations, including four with *BRAF* V600E mutations and one with G606R a mutation.^18^ Liu et al. ，Udo et al. also demonstrated *BRAF* V600E mutations in CMPTs.^10^, ^14^, ^16^ Other mutations have also been detected. Because mutations in oncogenes such as *EGFR* and *KRAS* have been shown to occur in lung adenocarcinoma, the presence of *KRAS* and *EGFR* mutations in CMPTs has been hypothesized to be associated with lung adenocarcinoma by some investigators.^7^, ^10^, ^14^ Although CMPT is usually regarded as a benign tumor, Miyai et al. proposed that CMPT with P53 alteration may be more likely to have malignant potential.^19^ Our case suggested that CMPT tends to be benign. However, this finding should be further validated.

In conclusion, CMPT is a rather rare and unique type of lung tumor. Because of the unclear mechanism of this disease, larger samples with longer follow‐up periods and further molecular mechanism investigation are necessary to determine the biological behavior of this rare lung tumor.

## CONFLICT OF INTEREST

All authors report no conflicts of interest.
